# Levels of different subtypes of tumour-infiltrating lymphocytes correlate with each other, with matched circulating lymphocytes, and with survival in breast cancer

**DOI:** 10.1007/s10549-020-05757-5

**Published:** 2020-06-23

**Authors:** Rashmi Verma, Andrew M. Hanby, Kieran Horgan, Eldo T. Verghese, Milene Volpato, Clive R. Carter, Thomas A. Hughes

**Affiliations:** 1 School of Medicine, University of Leeds, Leeds, UK; 2 Department of Breast Surgery, Bolton Hospital NHS Foundation Trust, Bolton, UK; 3 Department of Histopathology, St. James’s University Hospital, Leeds, UK; 4 Department of Breast Surgery, Leeds Teaching Hospitals NHS Trust, Leeds, UK; 5 Department of Transplant Immunology, Leeds Teaching Hospitals NHS Trust, Leeds, UK

**Keywords:** Tumour-infiltrating lymphocytes, TILs, Breast cancer, Estrogen receptor, Circulating lymphocytes

## Abstract

**Purpose:**

Breast cancer tumour-infiltrating lymphocytes associate with clinico-pathological factors, including survival, although the literature includes many conflicting findings. Our aim was to assess these associations for key lymphocyte subtypes and in different tumour compartments, to determine whether these provide differential correlations and could, therefore, explain published inconsistencies. Uniquely, we also examine whether infiltrating levels merely reflect systemic lymphocyte levels or whether local factors are predominant in recruitment.

**Methods:**

Immunohistochemistry was used to detect tumour-infiltrating CD20+ (B), CD4+ (helper T), CD8+ (cytotoxic T) and FoxP3+ (regulatory T) cells in breast cancers from 62 patients, with quantification in tumour stroma, tumour cell nests, and tumour margins. Levels were analysed with respect to clinico-pathological characteristics and matched circulating levels (determined by flow-cytometry).

**Results:**

CD4+ lymphocytes were the most prevalent subtype in tumour stroma and at tumour edge and CD8+ lymphocytes were most prevalent in tumour nests; FoxP3+ lymphocytes were rarest in all compartments. High grade or hormone receptor negative tumours generally had significantly increased lymphocytes, especially in tumour stroma. Only intra-tumoural levels of CD8+ lymphocytes correlated significantly with matched circulating levels (*p* < 0.03), suggesting that recruitment is mainly unrelated to systemic activity. High levels of stromal CD4+ and CD20+ cells associated with improved survival in hormone receptor negative cases (*p* < 0.04), while tumour nest CD8+ and FoxP3+ cells associated with poor survival in hormone receptor positives (*p* < 0.005).

**Conclusions:**

Lymphocyte subtype and location define differential impacts on tumour biology, therefore, roles of tumour-infiltrating lymphocytes will only be unravelled through thorough analyses that take this into account.

**Electronic supplementary material:**

The online version of this article (10.1007/s10549-020-05757-5) contains supplementary material, which is available to authorized users.

## Introduction

Tumour-infiltrating lymphocytes (TILs) are influential components of the tumour microenvironment and implications of their accumulation in breast cancers have been evaluated extensively [1, 2]. Extent of lymphocytic infiltration has been found to be associated with a variety of prognostic factors [3, 4], outcomes [5, 6] and, in some cases, outcomes after specific therapies [6, 7]. Despite this wealth of published data, key reproducible findings remain elusive, in part due to the range of methodologies used and variety of cohorts studied. In terms of methodologies, TILs were identified in many studies based on morphology using haematoxylin and eosin stained tissue, and indeed this was the recommendation by the International TILs Working Group [8]. However, this does not allow separate quantification of distinct TIL subsets, such as B and T cells, that may have differential recruitment to tumours and differential associations with prognosis [9]; the lack of this distinction is a potential cause of some conflicting findings in the literature. Accordingly, there is a growing literature describing specific TIL subtypes, using immunohistochemistry for detection of individual subtype-specific markers [10, 11], or recently multiplexed simultaneous imaging of subtypes [12]. A further difficulty is variation in methods for quantification of TILs and their locations. Some authors have quantified in descriptive terms only, such as defining infiltration as mild, moderate or heavy [13, 14], while others have assessed percentages of tissue area occupied by TILs [5, 6], or have counted individual cells [3, 10]. In terms of tissue regions, some have made no distinction between different tumour areas [3, 6], while others have limited their analyses to single regions, most commonly tumour stroma [4, 5, 7] for which quantification methods have been mainly standardised [8], or have quantified in a number of different regions separately [10, 11], for example in tumour stroma, closely associated with tumour cells, or adjacent to the tumour margin, although analysis of all these regions remains exceedingly rare.

The result of these differing methodologies is that summarising overall conclusions from the field is challenging, although reviews have been published [15, 16]. Some generalizable themes are: (1) There are typically more TILs in tumours showing markers of poor prognosis; (2) Higher levels of TILs are associated with better responses to neoadjuvant chemotherapy; (3) Different subclasses of TILs, such as cytotoxic T, helper T, or regulatory T cells, have different relationships with outcomes.

Here, we have taken a thorough approach of quantifying the presence, relationships between, and prognostic importance of four different subclasses of TILs (cytotoxic T, helper T, regulatory T, and B cells) in three separate tumoural compartments in a breast cancer cohort that we have characterised previously. In addition, uniquely, we have assessed how these levels correlate with the matched circulating levels of these cells, to infer whether TILs recruitment is tumour-driven independently of systemic levels – and if so for which TIL subtypes and in which tumoural compartments.

## Methods and materials

### Patient selection, ethics, clinical samples/data

Patient selection and recruitment has previously been described [17]. In brief, we recruited patients with diagnoses of operable primary breast cancer and scheduled to receive chemotherapy at Leeds Teaching Hospitals NHS Trust (LTHT) between June 2011 and January 2012. Written, informed consent was taken. Exclusion criteria included age > 75 years, long-term steroids/immunosuppressive drug use, previous breast cancer, and previous chemotherapy within 10 years. Ethical approval for patient recruitment, data collection, and subsequent analyses was obtained from Leeds East REC (references 06/Q1206/217 and 06/Q1206/180). Data regarding circulating lymphocytes in these patients has already been published [17]; circulating lymphocytes data used here are the pre-chemotherapy values, which were assessed at the closest time-points to the point of tissue sampling (biopsy or resection as appropriate). 62 patients from this previous study have been included in this new work, based on tissue availability; clinico-pathological data for these patients are shown in Table 1. Survival time was defined as time from diagnosis to death from cancer or to last alive follow up. Data are reported in accordance with REMARK [18].

**Table 1:** Clinico-pathological details of the cohort (*n* = 62)

Characteristic	*N* (%)
Age	
20–40 years	4 (6.5%)
41–60 years	38 (61.2%)
61–75 years	20 (32.3%)
Tumour grade	
Grade 1	0 (0%)
Grade 2	25 (40.3%)
Grade 3	37 (59.7%)
Tumour size	
< 2 cm	34 (54.8%)
2–5 cm	26 (42.0%)
> 5 cm	2 (3.2%)
Nodal metastasis	
No	30 (48.4%)
Yes	32 (51.6%)
Hormone receptor status	
ER/PR positive	35 (56.4%)
ER/PR negative	27 (44.0%)
Her2 status	
Negative	51 (82.2%)
Positive	11 (17.7%)
Chemotherapy type	
Neoadjuvant	7 (11.3%)
Adjuvant	55 (88.7%)
Chemotherapy regimen	
EC/FEC	29 (46.8%)
EC+ GCSF/EC+ DOCET+ GCSF	31 (50.0%)
Paclitaxel/others	2 (3.2%)
Radiotherapy	
No	10 (16.1%)
Yes	52 (83.9%)

*EC* epirubicin/cyclophosphamide, *FEC* fluorouracil/epirubicin/cyclophosphamide, *GCSF* granulocyte colony-stimulating factor, *DOCET* docetaxel

### Immunohistochemistry (IHC)

Archival tumour blocks were retrieved from the LTHT pathology department: pre-treatment diagnostic biopsies for cases treated with neoadjuvant chemotherapy (*n* = 7), surgical resections for the remainder. Hormone and HER2 status of the neoadjuvant cases are shown in Table S1. 5 μm sections were taken onto SuperFrost plus slides (Menzel-Glaser; Braunschweig, Germany). Sections were dewaxed (xylene) and rehydrated (ethanol) before washing in running tap water. Antigen retrieval was performed in 10 mM citric buffer (pH 6.0) heated by microwave (full power) for 20 min. Slides were blocked in 3% hydrogen peroxide for 15 min followed by washes in tap water and Tris-Buffered Saline (TBS). Primary antibodies (Dako [Gostrup, Denmark] unless stated otherwise) were used in antibody diluent reagent (Invitrogen; Paisley, UK) as follows: anti-CD8 (clone C8/144B; 1:800 dilution; incubation overnight 4 °C), anti-CD4 (4B12; 1:200; 1 h 37 °C), anti-CD20 (L26; 1:400; 1 h 37 °C), anti-FoxP3 (236A/E7; Abcam [Cambridge, UK]; 1:400; overnight 4 °C). Slides were washed × 3 in TBS, and IHC was completed using anti-mouse Envision reagents (Dako; Gostrup, Denmark) following the manufacturer’s protocols. Slides were washed in running tap water (2 min), stained with Mayer’s haematoxylin (30 s), and then washed in running water (1 min), Scott’s water (1 min), and running water (rinse). Sections were dehydrated in ethanol (3 × 1 min) and xylene (3 × 5 min) before being mounted under coverslips in DePeX (VWR; Radnor, USA).

### Scoring of IHC staining

Slides were digitally scanned using ScanScopeXT at 20 × and were manually scored using Webscope (both Aperio; Vista, CA, USA). Scoring was performed in accordance with guidelines from the International TILs Working Group [8], applying these to IHC stains. Infiltration was quantified in three separate compartments: (1) intra-tumoural within tumour stroma; (2) intra-tumoural within tumour cell nests; (3) at tumour margin. Scoring protocols were designed in consultation with a consultant breast histopathologist (ETV). For scoring, entire slides were first examined at low magnification to assess broad distributions of tumour cells and TILs. 3 areas per resection or 2 for biopsies were then selected at ×10 magnification. The criteria used for selecting these areas included: presence of tumour cells, stroma and TILs, and lack of obvious staining artefacts or large areas of tissue loss. When TIL distribution was heterogeneous, areas with very high or low TILs were avoided. Selected areas were digitally marked at ×10 magnification using the Webscope pen tool, and marked areas were scored at ×20 magnification for TILs within the tumour nests and TILs in the tumour stromal areas. TILs within the tumour nests were defined as lymphocytes visually touching tumour cells, or within solid blocks of tumour cells without other visible stromal elements. These were quantified by counting the entire number of stained TILs and of tumour cells within the selected regions, and this was expressed as a continuous ratio of TILs to tumour cells (i.e. 0.1 indicates 1 TIL to 10 tumour cells). Stromal lymphocytes were assessed by estimating proportions of the stromal area occupied by lymphocytes and were expressed as percentages; these were estimated in increments of 5%, except when below 5% where individual integers were used. For each of these measures, means of the values for the multiple regions were taken as the values for each case. Finally, the tumour edge was located, when present on the slide, and TILs within this region was graded as mild, moderate or heavy infiltrate; edge was not scored on diagnostic biopsies from cases treated with neoadjuvant chemotherapy. Figures S1 and S2 contains representative images illustrating these scoring methods. All slides were scored by RV, while 10% of the slides for each antibody were additionally scored independently by breast histopathologist ETV, to allow statistical assessment of scoring reproducibility; scorers were blinded to histopathology data concerning the cases while scoring. Scatter plots of the independent scores are shown in Fig. S3, demonstrating a very high degree of concordance (Spearman’s correlation coefficients > 0.98, *p* < 0.001).

### Statistical analyses

Data were analysed using SPSSv22 (SPSS; Chicago, USA). Relationships were considered significant if *p* values were ≤ 0.05. For IHC analyses, association between two independent scorers was assessed using correlation coefficients. Wilcoxon’s signed rank test or Friedman’s two-way Analysis of Variance by Ranks (ANOVA) was used to assess differences in TIL distributions. Analyses involving the edge quantification of mild, moderate, and heavy were performed where appropriate by replacing these classes with numerical scores of 1, 2 and 3 respectively when appropriate. Correlation between different TIL locations, and with clinical and pathological factors were assessed using Spearman’s correlation coefficients. Survival analyses were performed using Kaplan–Meier plots with log rank statistics.

## Results

**Fig. 1:**
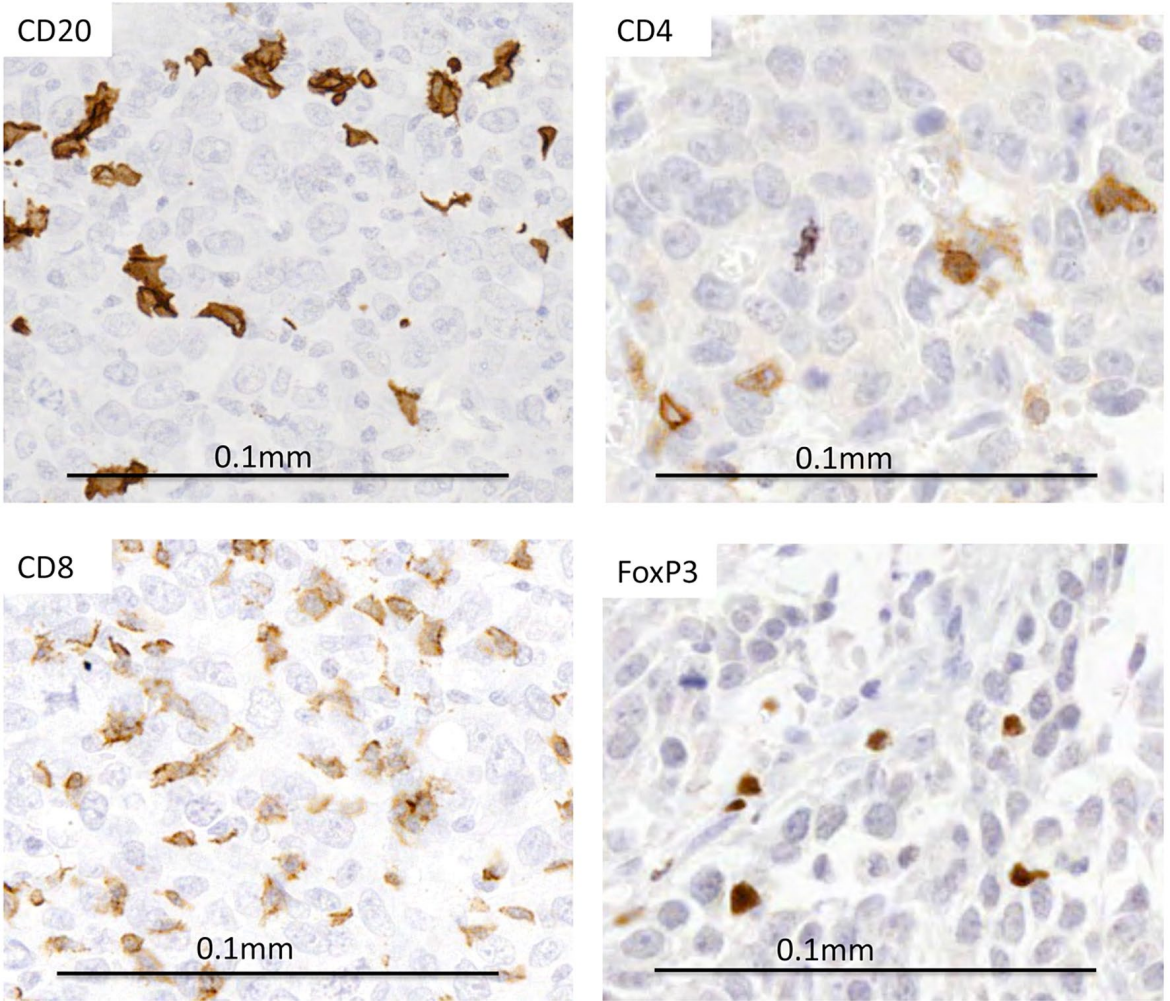
Representative images of immunohistochemical staining (brown) of markers CD20 (for B lymphocytes), CD4 (helper T lymphocytes), CD8 (cytotoxic T lymphocytes) and FoxP3+ (regulatory T lymphocytes) showing positivity within tumour nests

Building on our previous analyses of circulating levels of lymphocytes in primary breast cancer patients [17], we have now examined relative numbers and locations within breast cancer tissues of lymphocytes showing positivity for markers CD20 (B cells), CD4 (helper T cells), CD8 (cytotoxic T cells) and FoxP3 (regulatory T cells) in tumours from the same patients (*n* = 62). Clinico-pathological characteristics of patients included are listed (Table 1), while representative staining of lymphocytes is shown in Fig. 1. Lymphocytes were analysed in resection tissues for the majority of cases (*n* = 55), or using diagnostic biopsies for the cases treated with neoadjuvant chemotherapy (*n* = 7).

### Lymphocyte subtypes accumulate differentially in specific tumour locations

**Fig. 2:**
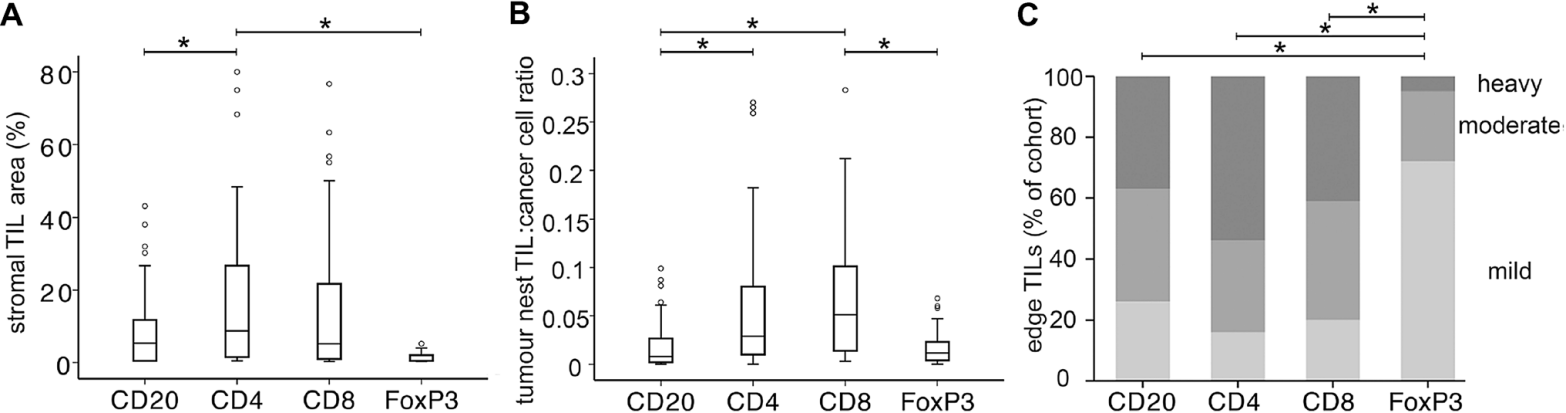
Tumour infiltrating lymphocytes differ in subclass in different intra-tumoural locations. Tumour sections from a cohort of 62 primary breast cancer patients were stained for lymphocyte markers CD20, CD4, CD8 or FoxP3. Tumour infiltrating lymphocytes (TILs) were quantified in three separate intra-tumoural locations: **a** within regions of tumour stroma (quantified as % of stromal area occupied by lymphocytes staining positive for marker under test); **b** within nests of tumour cells (quantified as the ratio of the number of lymphocytes staining positive for marker under test to the number of cancer cells); **c** tumour edge (classes as showing mild [light grey], moderate [mid grey] or heavy infiltration [dark grey] with lymphocytes staining positive for marker under test. Data in **a** and **b** are shown in box and whisker plots with mean value (horizontal bar), 50% of the data (box), interquartile range (whiskers) and outliers (circles). Data in **c** are shown as the proportion of the entire cohort showing each degree of infiltration. * indicates *p* < 0.05 by Wilcoxon’s signed rank tests

The four key lymphocyte subtypes above were quantified in three separate locations within the tumour microenvironment: in regions of tumour stroma, closely associated with tumour cells (in the tumour nests), and at the tumour edge (Fig. 2). Infiltration was often highly variable between different cancers; for example, the most extreme variation was seen with stromal CD4+ lymphocytes occupying from 0.5% to 80% of tumour stromal area. Within each location, distributions of scores for the four subtypes frequently differed significantly, demonstrating that the subtypes infiltrate tumours to different degrees. For example, CD20+ lymphocytes were significantly rarer than CD4+ lymphocytes in both stroma and tumour nests (*p* < 0.05). Interestingly, the most prevalent subtypes differed between locations, with CD4+ lymphocytes being the most prevalent in tumour stroma and at the tumour edge, while CD8+ lymphocytes were the most prevalent in tumour nests. FoxP3+ lymphocytes were the rarest in all locations, demonstrating uniformly low levels in tumour stroma, more variable levels in the tumour nests levels although still relatively low, and predominantly only mild infiltration at the tumour edge. In order to support the validity of combining scores from resection samples with some from diagnostic biopsies, we have compared the distributions of stromal and tumour-nest scores from the combined cohort (*n* = 62) with those from only the resection samples (*n* = 55) (Table S2); no significant differences were found.

### Infiltration with lymphocyte subtypes positively correlates within and across tumour compartments, except T cell infiltration of tumour edge

Next, we tested whether infiltration with one lymphocyte subtype in a compartment was associated with infiltration with the other subtypes in the same compartment. The degree of infiltration of each subtype was significantly positively associated with infiltration of all other subtypes, and this was the case in all three compartments; Spearman’s correlation coefficients from 0.47 to 0.83 (*p* < 0.001) (Table S3). We also examined whether infiltration with a particular subtype in one compartment was associated with infiltration of this subtype into the other compartments (Table 2). Levels of CD20+ lymphocytes in each of the three compartments were significantly positively associated with each other, demonstrating that for this subtype the compartments are not strongly independent. For CD4+, CD8+ and FoxP3+ cells, there were also significant correlations between levels of infiltration in tumour stroma and tumour nests, but by contrast neither of these compartments showed significant correlations with infiltration at the tumour edge, suggesting that factors governing T cell infiltration differ between the intra-tumoural spaces and the tumour edge.

**Table 2:** Correlation between the different tumour compartments for each lymphocyte subtype

	Stromal % vs tumour nest ratio	Stromal % vs tumour edge	Tumour nest ratio vs tumour edge
CD20	0.593 (*p* < 0.001)	0.304 (*p* = 0.017)	0.338 (*p* = 0.008)
CD4	0.694 (*p* < 0.001)	ns	ns
CD8	0.571 (*p* < 0.001)	ns	ns
FoxP3	0.470 (*p* < 0.001)	ns	ns

Spearman’s correlation tests were performed to assess associations between levels of lymphocytes in the tumour stroma, within the tumour nests and at tumour edge. Correlation coefficients are shown when there was a significant correlation with *p* values in brackets

*ns* not significant

### High grade or hormone receptor negative tumours have increased lymphocyte infiltrates, especially in tumour stroma

**Fig. 3:**
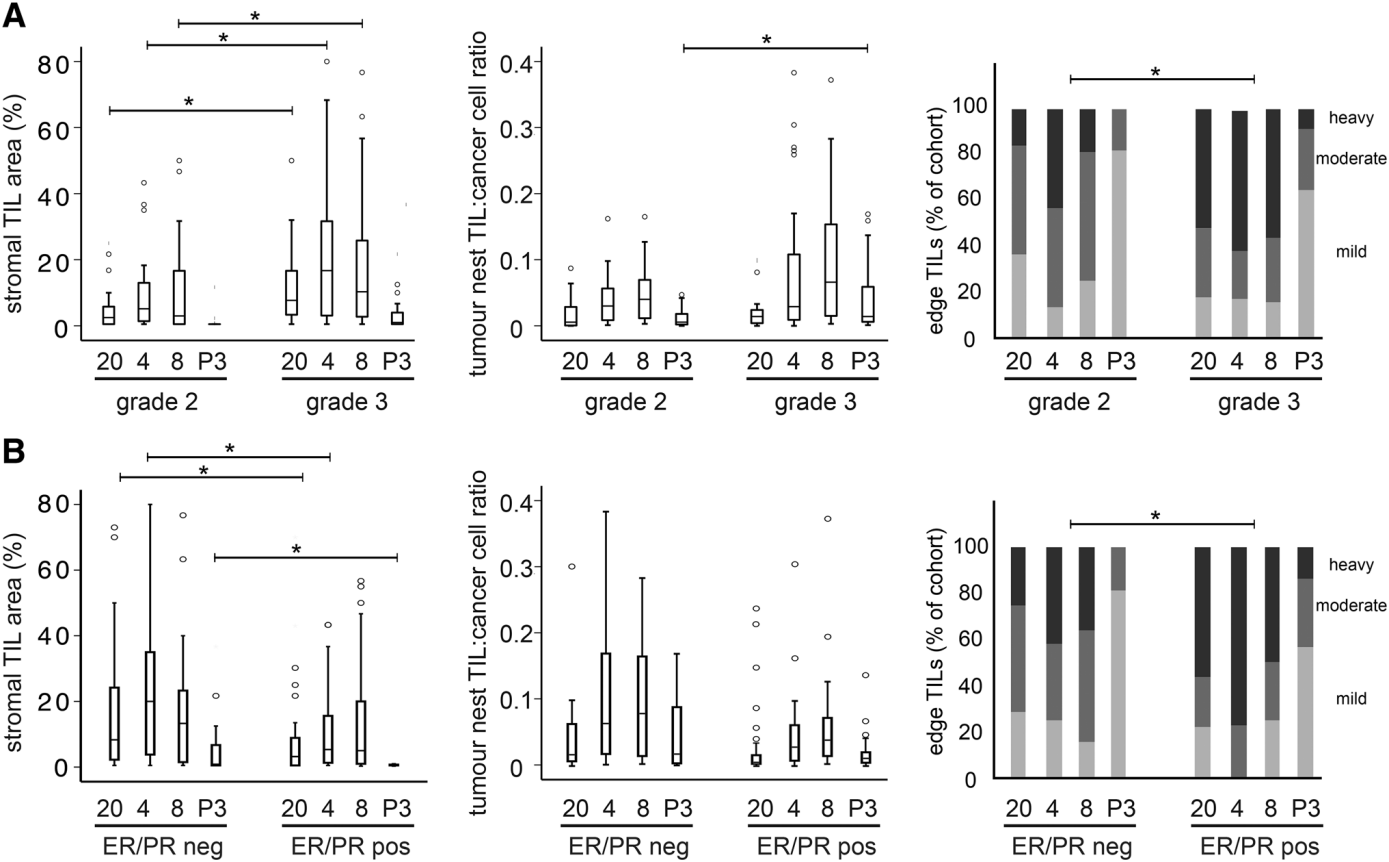
Tumour infiltrating lymphocytes differ based on tumour grade and hormone receptor status. Levels of lymphocyte staining for CD20 (20), CD4 (4), CD8 (8) or FoxP3 (P3) in either tumour stroma (left), tumour cell nests (middle), or tumour edge (right) were compared between tumours of grade 2 vs grade 3 (**a**) or ER/PR negative vs positive (**b**). * indicates *p* < 0.05 by Wilcoxon’s signed rank tests, except in the case of tumour edge where it indicates *p* < 0.05 by Friedman’s tests nm

TIL levels have previously been found to vary according to various prognostic features, including grade and receptor status [19–21]. We next tested whether this was the case for our cohort using the clinical data associated with cases (Table 1), with particular focus on whether correlations differed between separate tumour compartments. Our cohort did not contain any grade 1 tumours, as is typical for patients scheduled to receive cytotoxic chemotherapy, therefore we compared TIL infiltration between grade 2 and grade 3 tumours (Fig. 3a). Grade 3 tumours showed significantly greater infiltration of CD20+, CD8+ and FoxP3+ lymphocytes within the stromal compartment (all *p* < 0.05), while this was significant in the tumour nests only for FoxP3+ lymphocytes (*p* = 0.008). All four lymphocyte classes showed increased infiltration at the tumour edge in grade 3 tumours, as indicated by consistently increased proportions showing heavy infiltration. Furthermore, infiltrates were also significantly greater in ER/PR negative tumours compared to ER/PR positive (Fig. 3b). For example, CD20+, CD4+ and FoxP3+ lymphocytes were all significantly higher in the stromal compartment of the ER/PR negative tumours (all *p* < 0.03), and all four subtypes were similarly more prevalent at the tumour edge. By contrast, however, no subtypes differed significantly in the tumour nests (Fig. 3b). The other clinically relevant prognostic factors tumour size and nodal status did not show significant correlations with lymphocyte distribution. We concluded that poor prognostic factors that are direct cellular characteristics of the primary tumour are associated with increased TILs, and—surprisingly—that these associations are more prominent within tumour stroma and tumour edge as compared to the tumour nests themselves.

### Levels of intra-tumoural CD8+ lymphocytes correlate with levels of circulating CD8+ lymphocytes

Taking advantage of the fact that we also have available the matched circulating levels of different lymphocyte subtypes from a previous study [17], we tested to what extent TIL levels reflect systemic levels. Correlation tests were performed between the different compartments of CD20+, CD4+ and CD8+ TILs and the matched numbers of circulating CD20+, CD4+ and CD8+ cells. FoxP3+ TILs were compared with circulating levels of CD25+ FoxP3+, which is a more specific definition of regulatory T cells used for circulating cells as it is possible with flow-cytometric assessment. The only significant correlations were positive correlations between circulating CD8+ lymphocyte and CD8+ TILs levels in both the tumour stroma and tumour nests (*r* = 0.313 *p* = 0.024 and *r* = 0.375 *p* = 0.006, respectively). Interestingly, the tumour nest correlation was stronger in the ER/PR-negative tumours (*r* = 0.466, *p* = 0.033), but not significant in the ER/PR positive group. We concluded that levels of only CD8+ TILs correlated with matched circulating levels, suggesting that overall systemic lymphocyte levels are relatively weak influences on recruitment of most TILs, with local tumoural influences predominating.

### High levels of stromal CD4+ and CD20+ cells associate with improved survival only in ER/PR negative cases, while tumour nest CD8+ and FoxP3+ cells associate with poor survival only in ER/PR positive cases

Published data demonstrate that TIL levels correlate with survival of breast cancer patients [15, 16]. However, few studies have examined the relevance of different intra-tumoural locations for TIL subtypes, or in different cancer subgroups such as hormone receptor positive and negative. Kaplan–Meier survival analyses were used to compare survival in patients with either relatively high or low levels of each TIL subtype in each location. Receiver Operator Curve (ROC) analyses were used to define objective cut off values to dichotomise the cohort into these high and low groups for tumour nest TILs and tumour stroma TILs, based on sensitivity and specificity for prediction of cancer-specific death; cut off values are shown in Table S4.

**Fig. 4:**
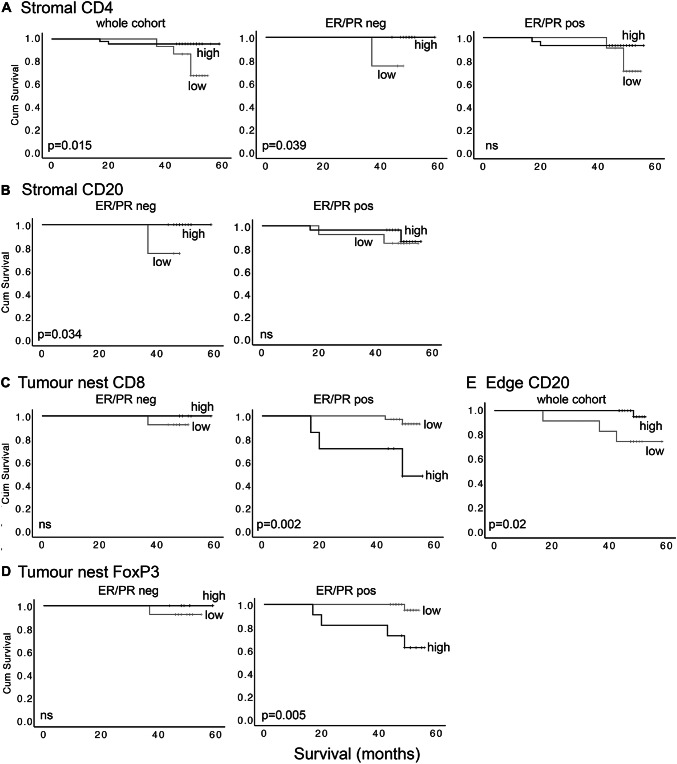
Tumour infiltrating lymphocytes predict outcome, depending on subclass, location, and ER/PR status of the cases. Levels of lymphocyte staining for CD4, CD20, CD8 or FoxP3 in tumour stroma, tumour cell nests or tumour edge were quantified and then dichotomised into high and low groups by receiver operator curve analyses or, in the case of edge, by combining heavy and moderate groups. The influence of levels on cancer survival was assessed using Kaplan–Meier survival analyses. **a** Stromal CD4 in total cohort (left), or the ER/PR negative (middle) and ER/PR positive (right) subgroups. **b** Stromal CD20 in ER/PR negatives (left) and ER/PR positives (right). **c** Tumour nest CD8 in the ER/PR negatives (left) and ER/PR positives (right). **d** Tumour nest FoxP3 in the ER/PR negatives (left) and ER/PR positives (right). **e** Tumour edge CD20 in the cohort for which edge scores were available (*n* = 46)

Using the whole cohort, the only significant relationship with survival was for stromal CD4+ lymphocytes, with high infiltration significantly associated with improved survival; these patients had mortality of 4% as compared to 36% in those with low infiltration at the median follow-up of 50 months (*p* = 0.015; Fig. 4a left panel). Interestingly, this correlation was based entirely on significance in ER/PR negative patients (*p* = 0.039), whereas there was no significant relationship in the ER/PR positive group (Fig. 4a middle and right panels). Similarly, high stromal CD20+ infiltration was also significantly associated with improved survival in ER/PR negative patients (*p* = 0.034), but not in the ER/PR positive group (Fig. 4b). Stromal CD8+ and FoxP3+ lymphocytes did not significantly correlate with outcomes. By contrast, in the tumour nests the CD8+ and FoxP3+ lymphocytes, and not the CD4+ and CD20+ cells, were significantly associated with survival, and only in the ER/PR positive patients (Fig. 4c, d). High levels of CD8+ or FoxP3+ lymphocytes were associated with reduced survival in ER/PR positive (*p* = 0.0013 and *p* = 0.005 respectively), but not ER/PR negative patients. We concluded that TIL subtypes were differentially associated with patient survival, and that these relationships depended on both intra-tumoural location, and hormone receptor status.

### ***High levels of CD20***+ ***TILs at tumour edge are associated with better survival***

Survival analyses were also performed to assess impacts of infiltration at tumour edge, using the three categories of infiltration already defined; mild, moderate and heavy. No lymphocyte subtypes showed significant impacts on survival, although higher levels of infiltration with CD20+ TILs showed a trend for being associated with improved outcomes (*p* = 0.054). However, this was significant when moderate and heavy infiltration groups were combined to allow comparison with mild infiltration (high vs low *p* = 0.02; Fig. 4e). Note that infiltration at tumour edge was only assessable in a cohort subset (*n* = 46), and in this context separate analysis in ER/PR positive/negative groups was not possible due to lack of statistical power.

## Discussion

TILs in breast cancer have been studied since the 1950s [22] and are now the subject of a vast literature. For the first few decades, reports supported the idea that TILs represented tumour immune recognition as a host defence mechanism, and accordingly their presence was associated with improved outcomes [23]. However, this simple story has not stood the test of time, with current reports detailing a huge variety of positive and negative associations with outcome or treatment response, depending on lymphocyte subtype, location within the tumour, or molecular subtype of the cases [1, 2, 15, 16]. Here, we have encompassed much of this complexity within one cohort in an effort to determine whether these factors are indeed associated with different conclusions, and therefore, that some literature that is superficially conflicting may in fact be concordant based on sometimes subtle differences in the location of the TILs quantified or the cohort case mix. The fact that we, and others, show TILs of different subclasses to infiltrate differentially between tumour compartments (Fig. 2) and in tumours of different pathological features (Fig. 3), supports the proposal that these factors may impact on tumour behaviour.

In terms of prognosis, we present some findings that conform to the model proposed in the last century with higher levels of TILs associated with improved outcomes, but we superimpose onto this complexity demonstrating that this is only the case for stromally located TILs that are positive for CD20 (B cells) or CD4 (helper T cells) and only within ER-negative cases. This is consistent with a published study in which stromal CD20 cells have been associated with longer survival in only the triple negative tumours (a majority subset of the ER-negative cases) [24], although this association has also been reported as holding irrespective of lymphocyte location and ER status [11]. Regarding CD4 cells, our observation is also in agreement with previous data showing the presence of these cells to correlate with longer survival specifically in ER-negative cases, although in this study lymphocytes were quantified throughout the tumour without analyses of separate compartments [25]. By contrast, we demonstrate that tumour nest located lymphocytes that are positive for CD8 (cytotoxic T cells) or FoxP3 (regulatory T cells) have exactly the opposite correlation with outcome, and only within ER-positive cases. There is, again, support for this in the literature in the relatively few studies that have separately quantified in tumour nest and stromal compartments. For example, in an overwhelmingly ER-positive cohort, tumour nest but not stromal CD8 cells were shown to correlate with reduced survival [26]. Similarly, FoxP3+ T cells have been shown to predict poor survival when located within the tumour nests but not in stroma that is at least one cell away from tumour cells, although this was not broken down on ER status [27], and the correlation has also been reported specifically in ER-positive but not in ER-negative cases, although this study failed to differentiate between tumour compartments [28].

A unique aspect of our study is that we examined whether levels of different TIL subtypes correlate with their matched circulating levels; to the best of our knowledge there are no such reports in breast, although we have found one recent study including only regulatory T cells in breast [29], and some in other cancers [30–32], with a largest cohort of 32. This is a key question because it informs our understanding of mechanistic influences, local or systemic, that drive accumulation of each TIL subtype in the separate compartments. It is also of potential clinical importance since in the event of a strong correlation, circulating levels could present an attractive minimally-invasive surrogate marker for TILs for use in prognostication. We found positive, although relatively weak, correlations between both stromal and tumour nest CD8+ cells, but not those at the tumour edge, and circulating CD8+ levels, but no significant correlations for any other lymphocyte type. This implies that tumour recruitment of B cells, helper T cells, and regulatory T cells is mainly governed by local factors irrespective of the systemic levels, whereas a component of recruitment of CD8+ cytotoxic cells results simply from reflecting levels available in the circulation. The conclusion regarding CD8+ cells is supported by a similar positive correlation in oral squamous cell carcinoma, although in this case CD4+ helper cells also correlated significantly [30], but contrasts with no correlation in cancers of the larynx [31] and a significant negative correlation in urothelial carcinoma recurrences [32]. Two reports comment on the correlation for T regulatory cells, both finding positive correlations, in cancers of the larynx [31] or in 17 cases of triple-negative breast cancer [29].

Overall, we conclude that TILs are recruited to different tumour compartments in the main by differential local influences that act with some degree of independence on the TIL subtypes. TIL levels reflect different tumour biologies in terms of their molecular subtype and prognosis. It is likely that thorough assessment of TIL subtype, location, and tumour molecular subtype will be required for TILs to deliver their potential as clinical prognostic or predictive markers.

## Electronic supplementary material

Below is the link to the electronic supplementary material.

Supplementary file 1 (PDF 1080 kb)
