# Supplementary material for: Levels of different subtypes of tumour-infiltrating lymphocytes correlate with each other, with matched circulating lymphocytes, and with survival in breast cancer
Source: Breast Cancer Res Treat. 2020 Jun 23;183(1):49–59. doi: 10.1007/s10549-020-05757-5 (PMC7376517; doi:10.1007/s10549-020-05757-5)
Supplement: Supplementary file 1 — (PDF 1080 kb) [file 10549_2020_5757_MOESM1_ESM.pdf]

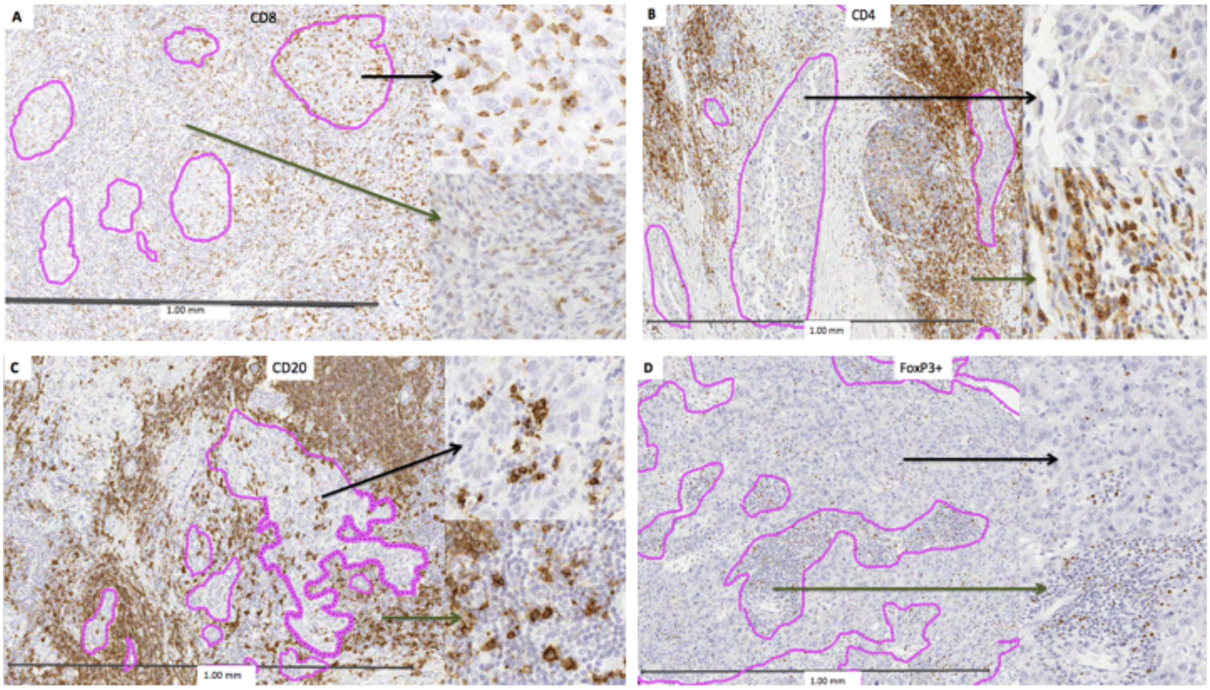

Figure S1. Tumour infiltrating lymphocytes were quantified as tumour cell interacting lymphocytes within the tumour cell nests, and as tumour stroma lymphocytes. Breast tumour sections were stained (brown) for CD8 (A), CD4 (B), CD20 (C) and Foxp3+ (D) allowing identification of cytotoxic T cells, helper T cells, B cells, and regulatory T cells respectively. Tumour cell islands are marked with pink in A, B, C. In D, stroma is marked in pink. Lymphocytes were counted within the tumour nests and quantified as a ratio relative to the number of tumour cells; the insets with black arrows show 10x magnification with lymphocytes (brown cells) in close proximity to cancer cells. Insets with green arrows show 10x magnification of tumour stromal regions in which lymphocytes (brown cells) were quantified as a percentage of the stromal area.

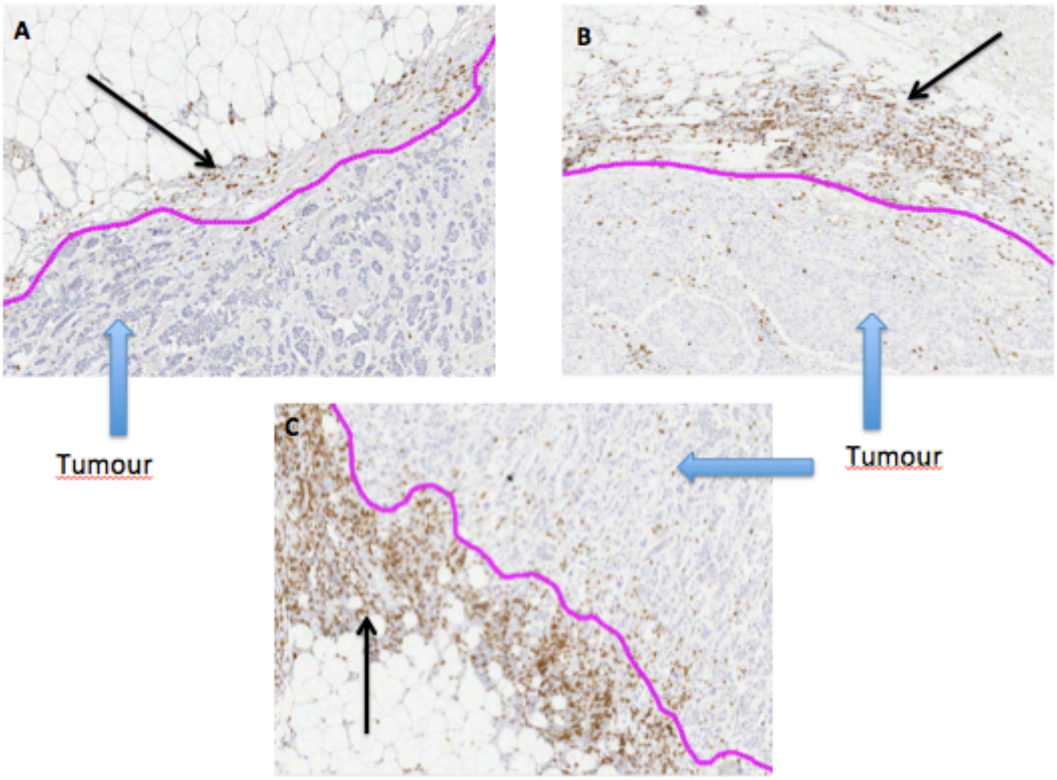

Figure S2. Lymphocyte infiltrate at the tumour edge was quantified. Breast tumour sections were stained (brown) for CD8 (cytotoxic T cells) in these representative examples. Tumour edge was located and the border is marked with a pink line. Infiltration immediately in this region was classed as mild (A) moderate (B) or heavy (C). Blue block arrows identify the tumour, and black arrows indicate lymphocytes (brown).

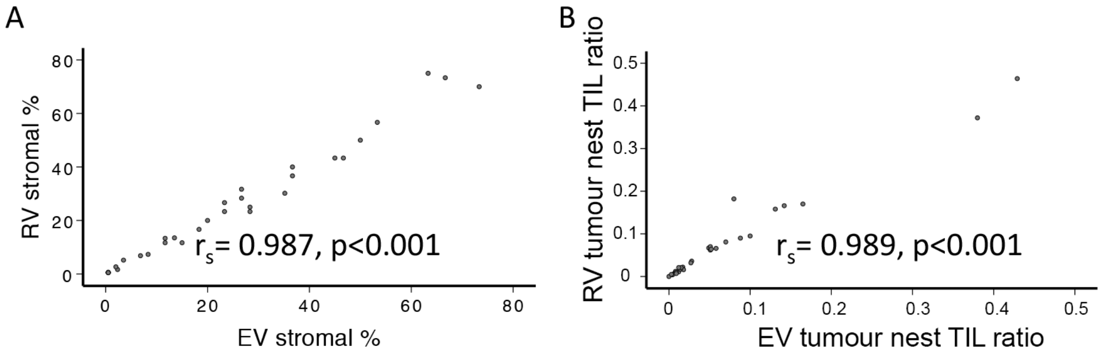

Figure S3. Assessments of lymphocyte prevalence were reproducible. Breast tumour sections representing 62 cases were stained for CD8, CD4, CD20 and FoxP3. 8 cases for each antibody were selected randomly and these were scored independently by two individuals (RV, and consultant breast histopathologist ETV) for the presence of tumour stromal lymphocytes (A) and lymphocytes within the tumour nests (B). Scatter plots are shown of scores from EV (x-axis) against RV (y-axis), along with Spearman's rho correlation coefficients (r_s) and p values.

Characteristic		n=7 (100%)
Hormone receptor status	ER/PR positive<br>ER/PR negative	3 (42.9%)<br>4 (57.1%)
Her2 status	Negative<br>Positive	7 (100%)<br>0 (0%)

Table S1. Receptor status of the cases treated with neoadjuvant chemotherapy (n=7).

	Compartment	CD20	CD4	CD8	FoxP3
**All (n=62)**	Stromal TIL area (%)	5.4 (12.85)	8.7 (25.3)	5.15 (20.7)	0.50 (1.55)
**ACT (n=55)**	Stromal TIL area (%)	6.0 (12.5)	8.7 (25.6)	8.3 (20.7)	0.5 (1.73)
**All vs ACT**		p=0.44	p=0.26	p=0.36	p=0.68
**All (n=62)**	Tumour nest TIL: cancer cell ratio	0.95 (2.72)	2.90 (7.55)	5.10 (9.28)	1.15 (2.0)
**ACT (n=55)**	Tumour nest TIL: cancer cell ratio	1.1 (2.5)	3.2 (7.7)	4.4 (8.7)	1.1 (2.08)
**All vs ACT**		p=0.91	p=0.88	p=0.85	p=0.54

Table S2. Tumour infiltrating lymphocytes levels do not vary significantly between the total cohort (n=62), or a reduced cohort excluding the 7 cases scored using biopsies (n=55). Tumour infiltrating lymphocytes (TILs) expressing CD20, CD4, CD8 or FoxP3 were quantified within regions of tumour stroma or within nests of tumour cells in breast cancer tissue from 62 patients. Medians (interquartile ranges) are shown for all patients (“all”) or for only those treated with adjuvant chemotherapy (“ACT”) and therefore scored using resection tissues. Distributions of values for all and ACT were compared using Mann Whitney U tests, with p values shown.

	Stromal %	Tumour nest ratio	Edge
CD20 vs CD4	0.649	0.604	0.774
CD20 vs CD8	0.505	0.593	0.728
CD20 vs FoxP3	0.568	0.622	0.657
CD4 vs CD8	0.585	0.734	0.827
CD4 vs FoxP3	0.626	0.688	0.751
CD8 vs FoxP3	0.472	0.694	0.751

Table S3. Levels of infiltration with one lymphocyte type are significantly associated with levels of infiltration of each other lymphocyte type. Spearman's correlation tests were performed to assess associations between levels of different lymphocyte types with each compartment (stroma, tumour nests, tumour edge). The table shows correlation coefficients in each case; all relationships were significant ($p<0.001$ in every case).

	Cut off values	
	Stroma %	Tumour nest ratio
CD20	0.9	0.0075
CD4	1.4	0.052
CD8	9.65	0.1125
FoxP3	0.6	0.019

Table S4. Cut off values were defined by Receiver Operator Curve analyses to dichotomise the cohort into high TIL and low TIL groups.
